# 172. Does Passive Reporting of *Streptococcus* spp Identified by Rapid Molecular Assay Change Antibacterial Prescribing Compared to Gram Stain (GS) Alone?

**DOI:** 10.1093/ofid/ofac492.250

**Published:** 2022-12-15

**Authors:** Adam W Archer, Heather L Cox, Lindsay E Donohue, Amy J Mathers

**Affiliations:** University of Virginia Health, Charlottesville, Virginia; University of Virginia Health, Charlottesville, Virginia; University of Virginia Health, Charlottesville, Virginia; University of Virginia Health, Charlottesville, Virginia

## Abstract

**Background:**

At University of Virginia Health, the detection of *Streptococcu*s spp via rapid molecular assay from blood cultures (BCx) was not historically reported in the EMR until the isolate was further identified. However this was reconsidered following anecdotal observation of inappropriate vancomycin (VAN) prescribing with GS alone. The purpose of this study was to investigate the impact of implementing passive reporting of *Streptococcus* spp on antibacterial prescribing patterns with a focus on VAN.

**Methods:**

This was a single-center, pre-post quasi-experimental study of adult inpatients with non-duplicate *Streptococcus* spp identified via rapid molecular assay (ePlex®; GenMark Diagnostics) from BCx before (pre-intervention, Jul 2020 – Sep 2021) and after (post-intervention, Oct 2021 - Apr 2022) implementing passive reporting of *Streptococcus* spp. The primary outcome was any antibacterial switch within 24 hours of GS. VAN days of therapy (DOT) within 7 days and rates of *Streptococcus* spp deemed contamination by the primary team were also assessed.

**Results:**

A total of 83 patients were included (58 pre- and 25 post-intervention). Baseline characteristics were similar including immunocompromised status (45% vs 52%), febrile neutropenia (28% vs 28%), and admission to a medical service (90% vs 96%). An antibacterial switch occurred within 24 hours of GS in 59% and 64% of the pre- and post-intervention groups, respectively, and was most commonly the addition of VAN (66% [25/38] vs 40% [8/20]; p=0.09). Fewer post-intervention patients continued VAN beyond 24 hours (66% vs 48%; p=0.15), but this was not statistically significant. The median VAN DOT remained unchanged at 2 days. A non-significant decrease in *Streptococcus* spp deemed contaminants was noted in the post-intervention arm (28% vs 16%; p=0.16). Finally, a non-streptococcal organism was isolated from concomitant BCx in approximately one-third of patients in each arm.

**Conclusion:**

Passively reporting *Streptococcus* spp identified by rapid molecular assay without paired stewardship intervention did not impact antibacterial prescribing. The identification of *Streptococcus* spp may be characterized by heterogeneous circumstances requiring nuanced real-time stewardship intervention.

**Disclosures:**

**All Authors**: No reported disclosures.

